# Immediate lipofilling in fat-augmented latissimus dorsi flap for breast reconstruction: a versatile and fully autologous option. Case report and technical considerations

**DOI:** 10.1097/RC9.0000000000000980

**Published:** 2026-09-14

**Authors:** Roberta Boffo, Stefano Uderzo, Bruno Lo Sapio, Mario Faenza, Alfredo Borriello

**Affiliations:** a Department of Plastic, Reconstructive and Aesthetic Surgery, Università degli Studi della Campania Luigi Vanvitelli, Napoli, Italy; b U.O.S.D. of Plastic Surgery, Ospedale del Mare, ASL Napoli 1 Centro, Naples, Italy

**Keywords:** autologous reconstruction, breast reconstruction, case report, FALD, immediate lipofilling, latissimus dorsi

## Abstract

**Introduction:**

The latissimus dorsi (LD) flap is a well-established option for breast reconstruction because of its reliable vascular pedicle and technical versatility. However, its main limitation is the relatively limited volume it provides, which has traditionally required the addition of a prosthetic implant to achieve adequate breast projection and symmetry. In selected patients with small- to moderate-sized breasts, the fat-augmented latissimus dorsi (FALD) flap may overcome this limitation by combining the LD flap with fat grafting.

**Presentation of case:**

We present the case of a patient who underwent a skin-sparing mastectomy with nipple–areolar complex excision. Immediate reconstruction was performed using an LD flap combined with intraoperative fat grafting. Fat harvested from the flanks was purified by decantation and injected during flap insetting into the mastectomy pocket. The postoperative course was uneventful, and the esthetic outcome was satisfactory, with good symmetry and volume preservation.

**Discussion:**

The FALD technique demonstrates significant reconstructive potential in selected cases, and performing lipofilling during the same operative session may also help reduce the need for staged revision procedures. This approach may also provide a secondary esthetic benefit by coupling breast reconstruction with body contouring. Despite its demonstrated advantages and favorable safety profile, FALD remains underutilized in current clinical practice.

**Conclusions:**

The FALD technique has proven to be a fully autologous, effective, and safe option for immediate breast reconstruction and deserves broader adoption, especially in cases in which implants or free flaps are not desirable or feasible.

## Introduction

The latissimus dorsi (LD) flap is one of the most established techniques for breast reconstruction because of its reliable vascular pedicle, technical versatility, and relatively straightforward execution^[^1^]^.

However, in its traditional form, the main limitation remains the volume it provides, which is often insufficient for complete breast reconstruction, especially in patients with medium-sized breasts. Historically, this limitation has frequently been addressed by combining the LD flap with a prosthetic implant or by extending the harvest of the surrounding subcutaneous tissue^[^1,2^]^.

More recently, fat grafting has emerged as an effective way to overcome this intrinsic volumetric limitation. The fat-augmented latissimus dorsi (FALD) flap represents an evolution of the traditional technique, combining the safety and reliability of the LD flap with the volumetric and contouring benefits of lipofilling^[^1,3^]^.

HIGHLIGHTS

- The fat-augmented latissimus dorsi (FALD) flap is a fully autologous and relatively straightforward technique that overcomes the intrinsic volumetric limitations of the LD flap without the use of implants.
- Immediate lipofilling during flap insetting enhances volume, projection, and contour, allowing tailored shaping of the reconstructed breast.
- Performing fat grafting in the same session may reduce the need for staged revision procedures and streamline the reconstructive pathway.
- Fat harvesting from donor areas such as the flanks or abdomen also provides a secondary esthetic contouring effect through simultaneous mini-liposuction.
- This case confirms the effectiveness, safety, and esthetic reliability of the FALD technique in selected patients with small- to moderate-volume breasts.

This approach allows surgeons not only to avoid the use of implants in selected patients but also to tailor the shape of the reconstructed breast more precisely, improving symmetry and overall esthetic harmony^[^3,4^]^.

Here, we present a clinical case treated with this approach in a patient with small- to moderate-sized breasts, where the reconstructive goal was to achieve a fully autologous reconstruction while avoiding both microsurgical techniques and prosthetic implants, and in whom a standard LD flap alone was unlikely to provide sufficient volume.

This work has been reported in line with the SCARE criteria.

## Presentation of case

A 68-year-old non-smoking woman with no significant medical comorbidities, a BMI of 23 kg/m^2^, and small- to moderate-sized breasts based on clinical assessment (Fig. 1) underwent a left skin-sparing mastectomy with nipple–areolar complex excision for the treatment of breast cancer. Final histopathology revealed a pT2N3 luminal B HER2-negative invasive ductal carcinoma.

**Figure 1.**
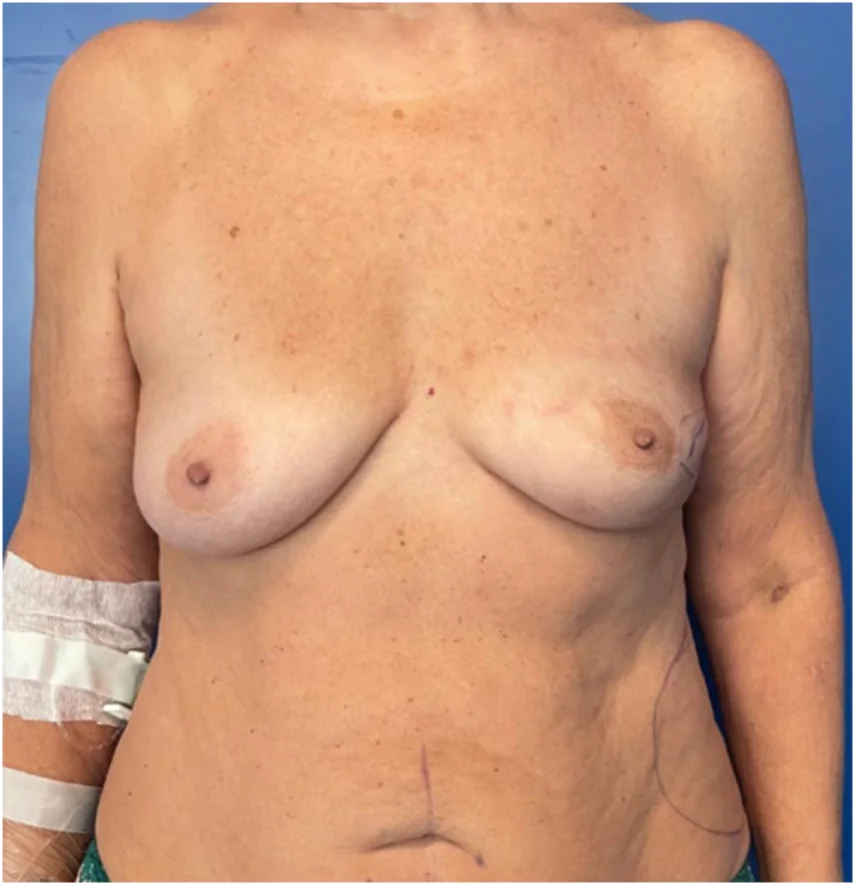
Preoperative condition.

Reconstruction was performed simultaneously using an LD flap augmented with immediate fat grafting.

The aim of our immediate reconstruction was to obtain a natural neobreast that was as symmetric as possible to the healthy contralateral side, without resorting to implants or free flaps, and without performing any procedure on the unaffected breast.

The LD flap was harvested along an oblique axis (Fig. 2), rotated 180°, and transferred into the mastectomy pocket through an axillary tunnel, leaving the donor-site scar concealed by the bra line.

**Figure 2.**
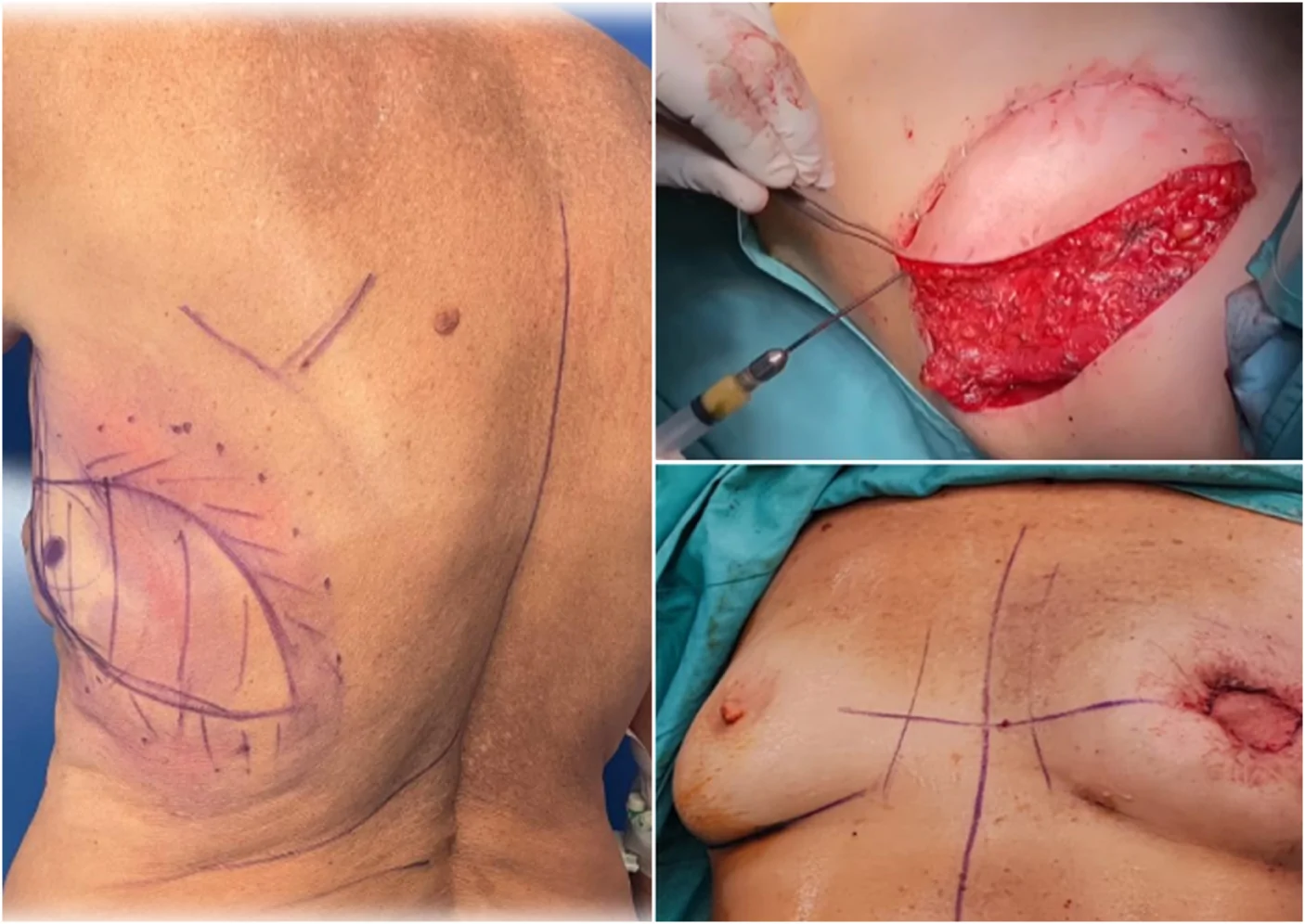
Intraoperative views (skin marking, fat grafting, and immediate postoperative result on the operating table).

Adipose tissue was harvested from the flanks during the mastectomy, purified by decantation with removal of the oil and fluid fractions, and grafted into the flap during inset using a multisite, multilayer technique. Small aliquots of fat were distributed throughout well-vascularized tissues to maximize the graft-to-recipient interface and optimize graft survival. Approximately 120 cc of fat was injected (Fig. 2).

The skin paddle was partially de-epithelialized and tailored to reconstruct the nipple-areola complex defect. The inframammary fold was redefined using internal 2-0 Monocryl sutures to improve lower pole contour and symmetry.

Cutaneous closure was completed with absorbable monofilament intradermal sutures. Two drains were placed, one in the mastectomy pocket and one at the donor site.

The postoperative course was uneventful. The drains were removed on postoperative day 4 (breast) and day 6 (donor site), and the patient was discharged on day 6. Postoperative care included regular outpatient follow-up, application of fatty gauze dressings, and suture removal on day 15.

The esthetic result showed stable volume, good projection, and satisfactory symmetry from the immediate postoperative period onward, with an appearance very similar to the preoperative breast shape. At 6 months postoperatively, the esthetic outcome remained highly satisfactory, and no refinements were required (Fig. 3).

**Figure 3.**
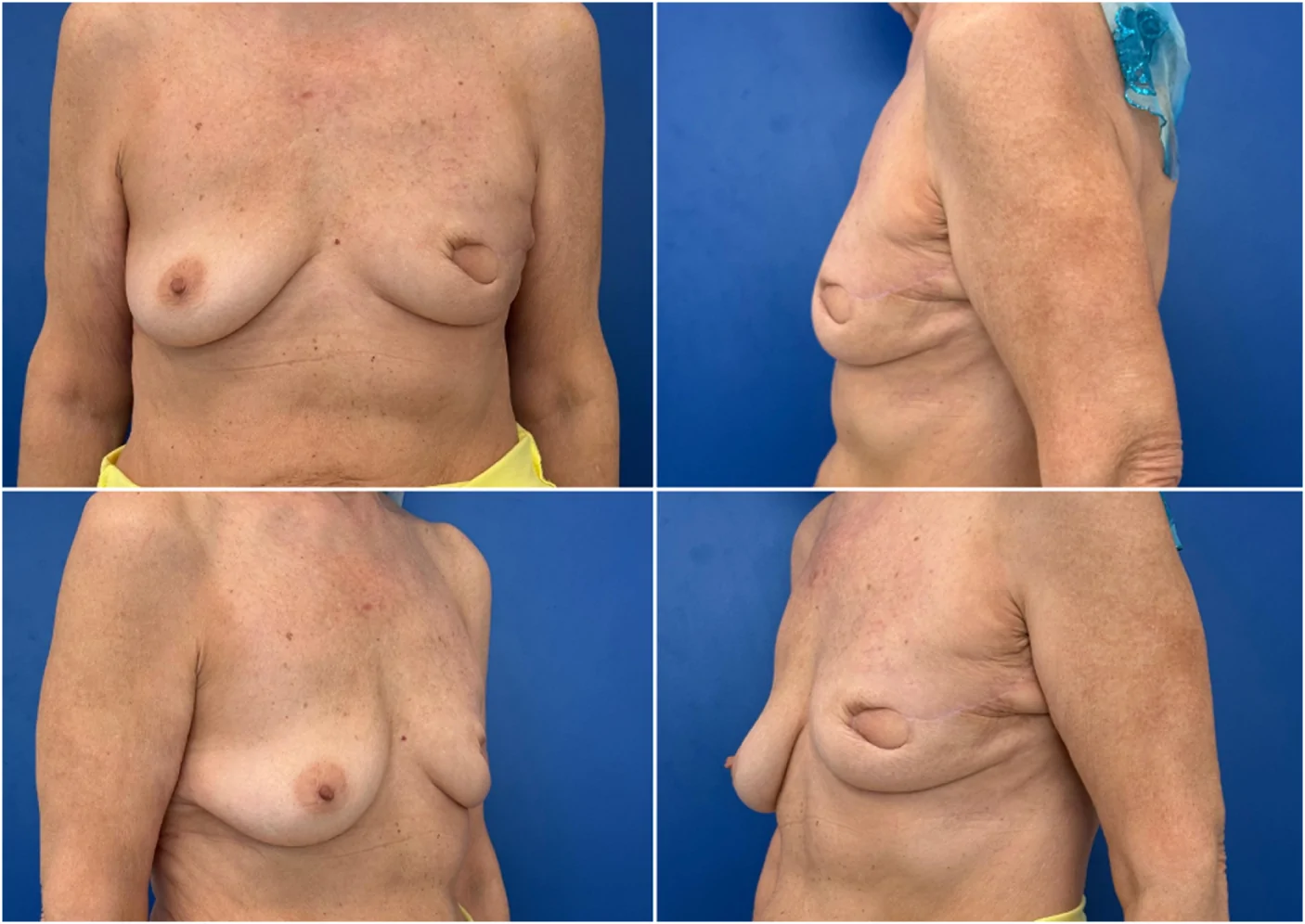
Postoperative results at 6 months.

The patient reported high satisfaction.

Completion of the reconstruction is planned in a single future session, including reconstruction of the nipple-areola complex with a local flap, scar revision, and additional lipofilling if required.

No contralateral symmetrization is planned in accordance with the patient’s preference to avoid additional procedures on the unaffected breast.

## Discussion

The FALD represents a significant evolution of the traditional LD flap, especially in cases where free flaps are not an option or implants are to be avoided^[^1,2^]^.

Unlike hybrid reconstructions involving prosthetic implants, the FALD technique allows for a fully autologous approach, combining the safety and simplicity of the thoracodorsal pedicle with the volumetric and contour-enhancing benefits of fat grafting.

Several authors have reported the effectiveness of this approach in both immediate and delayed settings, particularly in slim patients or those with moderate breast volume, where the muscle alone is insufficient to ensure satisfactory symmetry^[^3,4^]^.

Although the FALD flap was first described by Santanelli di Pompeo *et al* in 2014^[^5^]^ as an innovative and effective solution, its use in daily clinical practice remains relatively limited compared to other reconstructive options.

Importantly, the FALD technique has demonstrated a favorable safety profile. As reported by several authors, complication rates are comparable to or even lower than those seen with implant-based methods^[^6^]^. Furthermore, in irradiated patients undergoing delayed breast reconstruction, the FALD flap has been associated with a lower rate of breast-related complications compared with implant-based LD reconstruction^[^7^]^.

Finally, FALD has also proven to be a reliable salvage option after implant-based reconstruction failure, allowing safe conversion to a fully autologous reconstruction with low major complication rates, even in previously irradiated patients^[^8^]^.

Performing fat grafting during the same operative session offers several advantages over delayed grafting. It increases flap volume during inset, improving projection, contour, and symmetry while allowing immediate shaping of the reconstructed breast^[^9^]^. In addition, it may reduce the need for secondary revision procedures, although further lipofilling sessions may still be required in selected cases^[^9^]^. The single-stage approach may therefore decrease the procedural burden for both the patient and the healthcare system, while also providing the additional esthetic benefit of simultaneous liposuction.

In our case, fat grafting proved a valuable adjunct that enabled us to shape the breast profile to the point that contralateral symmetrization was unnecessary. The goal, which was achieved, was a natural and symmetric reconstruction in a single stage, avoiding further procedures on the healthy breast in accordance with the patient’s wishes.

Another key aspect of the FALD technique is the timing and site of fat injection during flap transfer. Several authors have shown that immediate grafting to a freshly harvested flap with multilayer infiltration of the LD muscle, pectoralis major, and mastectomy flaps can be performed safely while maximizing volume transfer^[^10^]^. Alternative technical approaches, such as primary fat grafting to the pectoralis major muscle^[^11^]^ or fat transfer before flap disinsertion^[^12^]^, have also been described, highlighting the versatility of immediate fat grafting strategies during LD breast reconstruction.

An additional practical advantage of the FALD technique is the possibility of harvesting fat from typical areas of accumulation, such as the flanks or abdomen, at the time of mastectomy, which may also provide a secondary esthetic benefit, as patients often appreciate the opportunity to improve their body contour through simultaneous mini-liposuction. Although ancillary, this aspect contributes to patients’ psychological well-being during what is often a physically and emotionally demanding surgical journey.

Although FALD reconstruction offers several advantages, it is not without complications and limitations. As with traditional LD flap reconstruction, donor-site seroma remains one of the most frequently reported complications. In addition, the fat grafting component introduces specific considerations, including the possibility of oil cyst formation, fat necrosis, and the inherent unpredictability of fat resorption^[^1,3^]^. Nevertheless, comparative studies have reported favorable outcomes for FALD reconstruction when compared with implant-based LD reconstruction. While donor-site morbidity appears comparable, FALD avoids implant-related complications such as capsular contracture, implant extrusion, rupture, and the potential need for future implant replacement, while maintaining the advantages of an entirely autologous approach^[^6,7^]^. Compared with DIEP flap reconstruction, FALD avoids microsurgical anastomoses and may reduce operative complexity and operative time. On the other hand, DIEP reconstruction preserves the LD muscle and generally provides greater volume, making it more suitable for larger breast reconstructions. Conversely, FALD may represent an attractive option in patients with small- to moderate-sized breasts or limited abdominal tissue availability, highlighting the importance of careful patient selection^[^13^]^.

Nevertheless, long-term volume maintenance remains one of the main challenges of this technique, as both muscle atrophy and partial fat resorption may occur over time, occasionally requiring secondary lipofilling procedures^[^1,3^]^.

Given these considerations, the main limitation of this report is the relatively short clinical follow-up currently available (9 months). Nevertheless, our findings are consistent with the favorable outcomes already reported in the literature.

## Conclusions

Given the favorable outcomes and numerous advantages described, we believe that the FALD with immediate fat grafting deserves broader consideration in everyday reconstructive practice.

Although the technique is well-established and relatively easy to perform, its use remains limited, with a continued preference for hybrid implant-based reconstructions or delayed fat grafting.

As confirmed by both the literature and our experience, FALD represents an effective, safe, and highly satisfactory option that should not remain confined to select cases, but rather be considered more frequently among first-line reconstructive options in selected patients, particularly those with small-to-moderate breasts in whom implants or free flaps are not desirable or feasible.

## Data Availability

Not applicable.
